# The role of perceived social norms in non-suicidal self-injury and suicidality: A systematic scoping review

**DOI:** 10.1371/journal.pone.0286118

**Published:** 2023-06-23

**Authors:** Robert C. Dempsey, Sophia E. Fedorowicz, Alex M. Wood

**Affiliations:** 1 Department of Psychology, Faculty of Health and Education, Manchester Metropolitan University, Manchester, United Kingdom; 2 School of Psychology and Therapeutic Studies, Faculty of Health and Social Sciences, Leeds Trinity University, Leeds, United Kingdom; Universidade Federal do Rio Grande do Sul, BRAZIL

## Abstract

Social norms are an important influence on health-related behaviours and intention formation. As both suicidal behaviour and non-suicidal self-injury (NSSI) can be motivated by intentions, perceived social norms may have an important role in suicide and NSSI outcomes, although no existing reviews of this association exist. Following the PRISMA Scoping Review extension guidance, a scoping review based on systematic searches of key databases was conducted to identify published English language studies investigating the role of perceived social norms in suicidality and NSSI. Information regarding the types of social norms studied, their relationship to suicidality/NSSI outcomes, study samples and designs was charted. Thirty-six eligible studies (31 quantitative, 4 qualitative, 1 mixed methods) sampling various populations across mostly non-clinical settings were identified and narratively synthesised. Studies varied in how social norms were operationalised, measured, and investigated/explored. Most studies focused on the role of conformity to perceived masculine social norms or to some form of subjective, descriptive, or injunctive norms; there were limited studies on female/feminine norms, pro-social/protective norms, or broader gender/sexuality norms. Most studies (n = 31) were cross-sectional (quantitative) in design, few were based on existing theories of suicide/NSSI or social norms, and none concurrently tested theories of social norms and NSSI/suicidality. Perceived social norms and stronger conformity to norms were generally associated with worse NSSI/suicidality, although some pro-social norms appeared to be protective (e.g., perceived parental norms for adolescents). Whilst conformity to restrictive perceived social norms may be related to poorer suicide and NSSI outcomes, there is a lack of consistency in the literature in how social norms are defined and measured, a lack of theory-based hypothesis testing, and few longitudinal studies. There is a need for more nuanced, theory-based, investigations of how, when, where, why, and for whom, perceived norms have a causal role in NSSI and suicidality outcomes.

## Introduction

The idea that social norms, specifically perceived social norms, may be causal factors in suicide and non-suicidal self-injury (NSSI) dates back to at least the late-1800s [1, 2]. Social norms are widely studied as determinants of health-related behaviours but are subject to inconsistent definitions and conceptualisations in the research literature [3, 4]. Social norms may encapsulate an individual’s perceptions of the majority of others’ behaviours (descriptive norms) and approval of behaviours (injunctive norms), to perceptions of what important individuals do and think [3–5]. There remains little systematic psychological research into the types of social norms associated with NSSI or suicidality, or how non/conformity to perceived norms exacerbates risk or protects an individual against harm. We conducted the first scoping review to understand the extent of the literature investigating the relationship between NSSI and suicidality with social norms, specifically focusing on identifying the types of perceived social norms studied in relation to NSSI and suicidality outcomes.

Suicide and NSSI are both global public health concerns but represent different phenomena motivated by different factors. Estimates of the prevalence of suicide and NSSI vary. For example, there are an estimated 700,000 to 1 million global annual deaths by suicide [6]. Variations are also noted for the prevalence of NSSI [7] with lifetime prevalence of NSSI of approximately 6% in adults [8] but higher prevalence rates reported amongst adolescents and younger adults (e.g., 13–17% [9]). Estimating the prevalence of NSSI is difficult due to different definitions of self-injury [10], yet self-injury irrespective of suicidal intent is one well-established risk factor for future suicidal behaviours [11]. There may exist some linkage between prior NSSI and future self-injury motivated by suicidal intent, especially through the repeated exposure and habituation to self-injury [12, 13]. There is growing recognition of the importance of intention formation in the experience of NSSI and suicidality, although they are differentiated in term of their underlying motivations and/or intent to die [14–18]. One key social influence on intention formation for a range of health-related behaviours are some form of (usually perceived) social norms [3, 19].

### Social influences on NSSI and suicidality

There is growing recognition of the importance of social factors in NSSI and suicidality-related outcomes. The clustering of incidences of NSSI and a number of deaths by suicide in specific locations and amongst specific social groups has led to the investigation of possible social influences on NSSI and suicidal behaviours [1, 20, 21], particularly peer influence amongst younger adolescents and young adults [22, 23]. Theories and concepts detailing the possible social origins of NSSI and suicidality include contagion [20], the Werther effect (relating to increases in suicide rates via media coverage) [24, 25], and suicide diffusion [26]. Although social factors and pressures may be implicated in NSSI and suicidality, purely focusing on group-level social factors may ignore important individual-level factors which exacerbate or protect against such external pressures.

Common to many psychological theories and approaches to understanding suicidality and NSSI is the influence of an individual’s perception of their social environment, such as their connectedness to others, their community, and social group [12, 27], and their status or rank within social groups [28]. For example, there is evidence of an increased risk of NSSI and suicidality amongst individuals who perceive that they have limited social support [29–33], who perceive themselves to be of low social rank [34], and who have been exposed to NSSI or a death by suicide in their peer group or family [21, 35]. Whilst theoretical models and empirical studies have highlighted the potential key role of individual perceptions of their social environment and relationships to others in suicidality, how perceptions of social norms are implicated in the pathways to NSSI and suicidality remains poorly understood.

### What are social norms?

Social norms are a key influence on personal behaviours and are broadly defined as “rules and standards that are understood by members of a group, and that guide and/or constrain social behaviour without the force of law” (p. 152) [36]. Social norms are proposed to influence intention formation for a range of behaviours and are featured in a number of prominent health-related behaviour models, most notably the Theory of Planned Behavior [4, 37]. Social norms are implicated in various health behaviours, including but not limited to: alcohol and other substance use [38, 39]; dietary behaviours [40, 41]; infection control behaviours [42, 43]; the use of contraceptives [44]; and use of sun protection [45]. As both NSSI and suicidality can be understood as similar health behaviours influenced by the formation of intentions, perceived social norms may have a similar influence on NSSI and suicidality-related outcomes.

Social norms have been defined, operationalised, and measured in multiple ways. Common operationalisations of social norms include the perceived rates of a behaviour (descriptive norms), perceived group attitudes or approval (injunctive norms) [46], subjective norms of an important other (the perceived social pressure to engage or not engage in a behaviour) [37], collective norms (“prevailing codes of conduct that either prescribe or proscribe behaviours that members of a group can enact”, p.129 [5]), moral norms (characterised by feelings of shame when violated [47]), to self-other discrepancies in terms of over or underestimating social behaviours and attitudes compared to actual norms [3, 4, 39, 48]. Social norms may also inform explicit legal frameworks and legislation based on agreed standards of permissible and unacceptable behaviour to more implicit and unwritten standards, such as widely shared beliefs about how individuals are expected to behave [49]. Social norms may operate at a societal or group level, based on social interaction and communication of expected standards amongst group members, or at an individual psychological level in the form of interpretations or perceptions of broader collective norms [5, 50]. There is, however, no universally agreed typology of social norms, and there are disagreements in the theoretical literature about the distinctiveness between different social norms types [47].

Perceived social norms may also serve different social influence functions. For example, perceived norms may indicate what to do in a given environment or context, especially novel situations, and thus encouraging conformity to match how others behave (“informational social influence”), to indicating what an individual ought or should do in terms of accepted standards or behaviours (“normative group pressure”) [51]. In relation to NSSI and suicidality, these different influences may be more nuanced compared to other behaviours. NSSI/suicidality are typically not public or majority behaviours, which may not be typically viewed as socially acceptable, and so they may be viewed differently depending on the social context and the individual’s group affiliations.

### Social norms, NSSI and suicidality

There is an acknowledged lack of understanding on the relationship between social norms with NSSI and suicidality, with calls for further research and theory development on this possible relationship [22]. There have been specific calls for more work understanding the norms-suicidality/NSSI association amongst key high-risk groups who may be particularly influenced by perceived peer behaviours and attitudes (e.g., young people who identify with goth subcultures [52]). The differing conceptualisations of social norms presents a particular challenge for understanding their role in NSSI and suicidality, and health-related behaviours more broadly [3, 4, 50]. These different forms of social norms may potentially influence the development of NSSI and suicidal thoughts, feelings, and behaviours, in different ways. Social norms may act as a subtle social influence pressure to conform to a perceived set of standards [37] which may have the potential for penalties or social sanctions for non-conformity [49]. Deviation from actual collective norms, or a perception that one is deviating from social norms, could be important in precipitating and/or exacerbating suicidal thoughts and feelings, such as feelings of shame, humiliation, defeat and entrapment which have already been implicated in the psychological pathways to suicide [28, 53, 54]. Perceived social norms may have unique roles in terms of exacerbating NSSI and suicidality, as well as potentially being protective against poor outcomes depending on the type of social norms.

Although recent theoretical developments have focused on outlining the processes which may take an individual from thinking about NSSI and suicide towards planning and/or engaging in related behaviours [28, 55, 56], few theories explicitly describe the role of social norms in this process. Considering that social norms have been implicated in the formation of intentions to engage in a variety of protective and risky health behaviours, it is not unfeasible that social norms have an influence on NSSI and suicidality, but where, when, and how social norms are implicated is unclear. To date, only one psychological theory of suicide (O’Connor’s Integrated Motivational Volitional, IMV, model [28, 57]) has outlined the possible role of social norms as one of a number of motivational moderators of the relationship between entrapment and suicidal ideation [28]. This proposed role of social norms has yet to be tested in the context of the IMV and there remains some lack of clarity of how social norms function as a moderator (as a buffering or exacerbating factor) or which types of norms are implicated in suicidal ideation or other outcomes. Whilst other psychologically-focused theoretical models of suicide and NSSI exist [55], these typically do not discuss the potential role of social norms in NSSI/suicidality outcomes.

This lack of clarity about the role of perceived social norms in NSSI and suicidality is problematic for the research literature and for the development of effective intervention approaches [22]. Social norms are a key component of many health behaviour change interventions and are typically featured in normative feedback comparing individual or group perceptions versus actual reported norms as a means to reduce the perceived social pressure to engage or not engage in range of behaviours (e.g., Social Norms Approach interventions reducing substance use) [3]. Due to the current lack of clarity about the types of social norms associated with NSSI and suicide-related outcomes, it is unclear whether normative feedback could be effectively used in harm prevention or harm reduction approaches or how such normative feedback should be phrased (i.e., which norms or reference groups to feature in feedback). Care is especially needed as normative feedback used with other health behaviours may not easily translate given the complexity of NSSI and suicide-related behaviours. For example, Social Norms Approach interventions typically highlight the actual, lower, rates of negative behaviours and peer approval versus the commonly held (mis)perceptions in order to promote behaviour change [3, 58, 59]. Given their relative infrequency, such feedback for NSSI/suicide-related behaviours might imply that such behaviours are more commonplace than the reality, thereby maintaining or increasing NSSI/suicidality. Alternatively, such normative feedback could further promote a sense of shame or difference amongst those who are suicidal or who have engaged in NSSI who deviate from the norm, leading to worse outcomes. Understanding the types of perceived social norms associated with NSSI and suicidality, and the directions of such relationships, would be important for understanding whether normative feedback could and/or should be used for NSSI/suicidality, and in developing appropriately targeted interventions.

### The present review

To date, there have been no prior reviews of the literature on the role of social norms in NSSI and suicidality-related outcomes, and no prior scoping of the types of perceived social norms studied in relation to NSSI/suicidality and the role of perceived social norms in these outcomes. Whilst NSSI and suicide have their differences as phenomena, both can be viewed as forms of health-behaviour influenced by the formation of intentions [55, 60], and such intentions may in turn be influenced by perceived social norms as a guide for how to behave and what others view as acceptable behaviours. There remains a lack of clarity in the research literature on how conformity or non-conformity to some form of perceived social norms is associated with NSSI and/or suicidality outcomes. The different, multiple, and often contrasting ways in which social norms have been defined presents a particular challenge for understanding their influence in health-related behaviours and intention formation more generally [3, 4], and in the development of appropriate behaviour change interventions.

The present review is the first attempt to scope the extent of the existing literature and identify the types of perceived social norms associated with NSSI and/or suicidality (i.e., suicidal thoughts, feelings, and behaviours). A scoping review approach [61] was adopted given the lack of prior reviews of social norms and NSSI/suicidality. A scoping review was appropriate considering existing acknowledgements of the heterogeneity of the social norms literature [3], specifically the inconsistent and often contradictory ways in which social norms have been operationalised in the broader literature (e.g., see Shulman and colleagues’ content analysis of studies employing social norms [4]). The present review is the first attempt to scope the types of social norms implicated in NSSI and suicidality outcomes and determine the extent of the existing literature and its methodological approaches, upon which future systematic literature approaches and syntheses may be conducted.

## Methods

### Design

A systematic scoping review [61] of the published research literature was conducted to identify the types of perceived social norms studied in relation to NSSI and suicidality, and the role of perceived social norms in these outcomes (see S1 File for the PRISMA Scoping Review Checklist). A scoping review was deemed appropriate based on a pilot review exercise which indicated the existence of a heterogenous literature on NSSI, suicidality, and social norms. In contrast to full systematic reviews, which tend to focus on specific research questions and specific study designs based on a narrow range of quality-assessed studies [61], scoping reviews are particularly useful where the extent of a literature is unknown or unexplored and can assist in mapping a literature and providing a broader overview of an evidence base regardless of its quality [62]. In line with recommendations for scoping reviews [61, 63], the present review represents an initial exploration of the literature, accommodates an identification of the types of perceived social norms implicated in NSSI and suicidality, as well as an understanding of the extent of the literature on this topic. The review protocol was registered on the Open Science Framework (see https://osf.io/btpzc/?view_only=11a12b31c7854225a022139b4228eed1).

### Eligibility criteria

Published studies were selected for inclusion based on the following criteria: (1) they investigated the role of perceived social norms in the individual experience of suicide-related thoughts, feelings and/or behaviours including self-harm or non-suicidal self-injury (e.g. as an outcome variable in quantitative studies or part of a research question or an identified theme for qualitative studies); (2) they constituted original empirical research; (3) were published in the English language; and (4) had been peer-reviewed. Original qualitative and quantitative studies were eligible for inclusion, whilst review articles or commentaries were ineligible. No restrictions in the ages or the nature of the study samples were applied to ensure the maximum inclusion of studies (e.g., adolescent, general population, and clinical samples). Studies on suicide attacks (e.g., suicide bombings, suicide terrorism), murder-suicides, or assisted suicide (i.e., euthanasia) were ineligible.

### Literature search strategy

Initial searches were conducted on the following databases in November 2020: Scopus, Web of Science, CINAHL, PubMed, PsychInfo, and ASSIA. Search terms related to ‘social norms’ and ‘suicidality’ or ‘NSSI’ focusing on the publication titles, abstract, keywords, and main text were used, for example: SOCIAL NORMS, NORMATIVE PERCEPTIONS, SUBJECTIVE NORMS, PERCEIVED NORMS, SUICID*, PARASUICIDE, and SELF-HARM. Broad search terms were used to capture a range of suicidality-related experiences (e.g., thoughts, behaviours, attempts) and the different operationalisations of “social norms” (e.g., norms, normative influence, normative misperceptions). For example, the following search string was used on the Web Of Science database with the results limited to English-language sources: TS = (suicid* AND “social norm*”). Searches were limited to English language articles where possible, but no other limits were applied to ensure the maximum inclusivity of returned articles for screening (i.e., no limit on date of publication or other filters were applied).

Studies which focused on reporting “normative scores” for psychometric assessments, or which analysed national trends data, were excluded as these did not specifically focus on individual perceptions of social norms and their influence on suicidality-related outcomes. Initial literature searches were restricted to articles published until the end of October 2020, with a top-up search conducted in June 2022 for articles published between November 2020 and May 2022. The lead author conducted the searches and screenings, with an independent check of the screenings and extracted data conducted by the second author, with disagreements between the authors and ambiguities in the reviewed studies discussed between the two authors. Hand searches of key suicidology journals which have published psychologically focused research (e.g., *Crisis*, *Archives of Suicide Research*, *Suicide and Life-Threatening Behavior*, *Journal of Affective Disorders*), together with searches of the publication records of prominent NSSI and suicidology researchers (e.g., Thomas Joiner, David Klonsky, Matthew Nock, Rory O’Connor, Ellen Townsend), were conducted to identify additional studies of relevance to the review.

### Data extraction and charting

Data extracted from eligible articles included: author name(s), date of publication and a full academic reference, sample characteristics (type/population, setting, sample sizes, screenings), methodology, analysis strategy, suicidality/NSSI measures, testing of suicide and/or social norms theory, primary and secondary findings (including indirect quantitative effects), evaluation and limitations of the study, and the conclusion(s) of the study.

## Results

### Summary of identified literature

**Fig 1:**
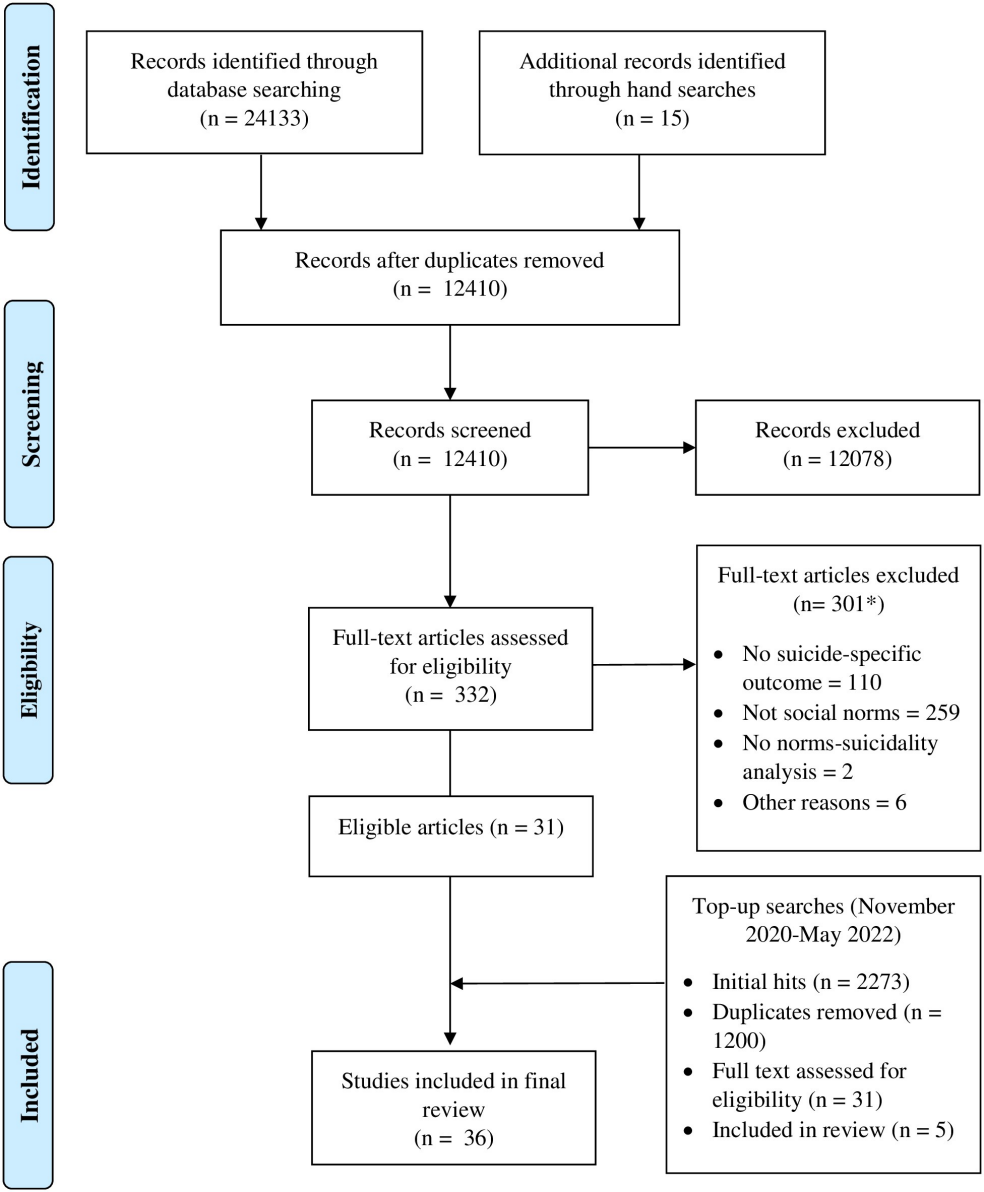
PRISMA scoping review flowchart detailing the screening process. * Note that studies could be excluded for not meeting both the suicidality/self-harm outcome and the social norms predictor/focus screening criteria.

Initial literature searches identified a sample of 31 eligible studies (4 qualitative, 26 quantitative, 1 mixed methods; see Fig 1), with an additional five quantitative studies identified through the top-up searches, making a final sample of 36 eligible studies (4 qualitative, 31 quantitative, 1 mixed methods).

Table 1 provides a summary of the study designs, social norms investigated or explored, and the suicidality and/or NSSI outcomes featured in the reviewed studies (S2 File includes additional details on study designs and methods). In terms of the characteristics of the reviewed studies, most (n = 28) were conducted in non-clinical settings (e.g., the community, schools, universities, and/or online participation panels), with six studies conducted in hospitals [64–69], one in a prison [70], and one study conducted at a US armed forces training base [71]. There was substantial variability in the sample demographics, particularly across participant ages, with studies sampling adolescents and high school students to older adults (see Table 1). Three studies were conducted in non-Western countries [72–74], with the majority of studies based in the UK, USA, Canada, Australia and New Zealand. Most studies used cross-sectional survey designs (n = 27), with five prospective self-report survey studies, and four studies featuring interview or focus groups (note that E. McDermott et al., 2018, featured both a cross-sectional survey and interviews [69]).

**Table 1:** Summary of the reviewed studies’ design, social norms investigated/measured, and the relationships between social norms and NSSI/suicidality.

Authors / Year	Sample	Design	Social Norms Investigated/Measured	Was a relationship between perceived social norms and self-harm/suicidality outcomes found?	
O’Connor & Armitage (2003) [65]	People admitted to a Scottish hospital after suicide attempt (n = 11) plus two control groups (33 hospital controls; 11 non-hospital controls)	Quantitative, cross-sectional survey	Subjective self-harm norm (TPB: perceived social pressure to harm oneself from perspective of ’people important to me’)	No	Subjective self-harm norms were not associated with intentions to self-harm in either correlational or regression analyses.
O’Connor et al. (2006) [64]	People admitted to hospital after a suicide attempt by overdose (Time 1 = 90; Time 2 = 23)	Quantitative. Prospective (3 month) self-report survey	TPB Descriptive Group Norms (perceptions of friends/peers’ self-harm behaviours and others’ attitudes of own self-harm) and Injunctive Social Norms (perceived important others’ approval of self-harm)	Yes	Positive correlations between injunctive/descriptive norms with past self-harm behaviours but not self-harm intentions. Significant descriptive norms by group identification interaction for self-harm intentions (those who more strongly identified with peers/friends had stronger self-harm intentions as perceived self-harm descriptive norms increased).
Skogstad et al. (2006) [70]	515 Male prisoners from six New Zealand prisons (variety of prison classes and offences)	Quantitative. Cross-sectional survey	Specific and general subjective norms based on the TPB (general = beliefs of people in the inmate’s life about seeking help; specific = seven influential referents for help seeking)	Yes	Positive (linear regression) associations between general and specific subjective help-seeking norms with intentions to seek help for suicidal ideation (specific norms the stronger predictor compared to general norms)
Pettingell et al. (2008) [86]	569 Urban American Indian adolescents aged 9–15 years	Quantitative. Cross-sectional survey	Perceived parental and peer prosocial behaviour norms	Yes	Perceived parental prosocial behaviour norms were negatively associated with history of suicide attempts for boys, but not girls. No effect for peer norms.
O’Connor et al. (2009) [87]	2008 15–16-year-old high school students in Scotland	Quantitative. Cross-sectional survey	Peer and friends ’group norms’ (perceived attitudes towards self-harm) from a previous study [65]	Yes	Perceived peer group norms (self-harm attitudes) positively associated with increased odds for lifetime self-harm for boys, not girls. Positive associations between lifetime self-harm by friends (girls only) and family (boys and girls)–but unclear if these were norms measures.
Swahn et al. (2010) [88]	87349 Middle and High School students in Georgia, USA (Grades 6, 8, 10, 12; ages 11–18 years)	Quantitative. Cross-sectional survey	Perceived peer and adult disapproval of alcohol use (injunctive norm)	Yes	Perceived friend and adult alcohol disapproval norms were negatively associated with past year suicide attempts. Some sex-specific effects including: a negative relationship for adult norms with boys’ suicide attempts, and friend norms with girls’ suicide attempts. Moderated effects of personal alcohol use and suicide attempts history by perceived peer and adult disapproval. Lower suicide risk amongst those who did not use alcohol but thought family/peers disapproved of alcohol use, higher risk amongst those who used alcohol and thought friends disapproved of use.
Pisani et al. (2012) [89]	2737 US high school students aged 14–17 years (12 schools)	Quantitative. Cross-sectional survey	Perceived acceptability of seeking help for emotional distress (from perspective of friends and family)	Partly	More positive perceived help-seeking acceptability norms at school were associated with higher odds for disclosing suicidal ideation and seeking help–but only in single univariate models (not in multivariate models alongside other psychosocial variables).
Easton et al. (2013) [75]	487 US Men (aged 19–84 years) recruited via support groups for those who had been sexually abused in childhood (CSA)	Quantitative. Cross-sectional survey	Conformity to masculine norms (CMNI-22)	Yes	Stronger conformity to masculine social norms were associated with increased odds of history of suicide attempts (past 12 months).
Jordal et al. (2013) [72]	28 Unmarried/single Sri Lankan pregnant women (third trimester) or who gave birth in past year, ages 15–33 years	Qualitative (content analysis). Cross-sectional semi-structured interviews	Perceived cultural and societal norms of expectations of women and childbirth (i.e., giving birth to children when married)	Yes	Qualitative data: norms discussed in themes relating to violating perceived sociocultural expectations of motherhood by giving birth outside of marriage
Geisner et al. (2015) [85]	1577 US university students aged 18–24 years (single university)	Quantitative. Cross-sectional survey	Perceived same-campus student mood norms (percentage of students feel sad, depressed or suicidal in past two weeks) compared with actual reported norm (students then categorised into over-, under-estimating, or correct estimators)	Yes	Positive association between perceived peer suicidal ideation with personal/own suicidal ideation (over past two weeks).
Granato et al. (2015) [77]	551 US university students (single university)	Quantitative. Cross-sectional survey	Conformity to masculine role norms (Gender Role Conflict Scale)	Yes	Direct effects of conformity to masculine social norms (Success, Power and Competition; Restrictive Emotionality; Restrictive Affectionate Behaviour between Men) with the acquired capability for suicide. These relationships were mediated by painful life events (not for the Restrictive Affectionate Behaviour between Men norms; but for the Conflict between Work and Family norms). Participant sex did not moderate these mediated effects.
Coleman & Paggi (2017) [90]	110 US adults aged 60 years and older, and their family/partners (who rated participants on the same measures)	Quantitative. Cross-sectional survey	Conformity to traditional masculine social norms (MRNI-R)	No	No significant relationship between masculine social norms with either suicidal ideation or behaviour.
Hassett & Isbister (2017) [68]	8 British Young men (16–18 years) in contact with mental health services due to self-harm	Qualitative (IPA). Cross-sectional semi-structured interviews	Conforming to and changing perceived masculine social norms	Yes	Qualitative data: themes discuss how the male participants (with histories of suicidal thoughts/behaviours) perceived masculine norms in relation to other young men (specifically conforming to muscularity/self-reliance) and how changing these norms promoted help-seeking.
Oliffe et al. (2017) [91]	20 Canadian men aged 20–62 years with lifetime histories of suicidal thoughts and/or plans/attempts	Qualitative (Constant Comparative Methods). Photo-elicitation interview	Mixture of perceived masculine norms (control, self-reliance) and perceived general social norms about seeking help and being suicidal or depressed	Yes	Qualitative data: norms discussed as part of several themes, particularly recovery from injury and having ongoing struggles, especially conforming to masculine norms of strength/silence in the context of suicidality and illness.
Pirkis et al. (2017) [92]	13884 Australian men (aged 18–55 years)	Quantitative. Cross-sectional survey from first wave of a larger scale multi-wave study	Conformity to masculine norms (CMNI-22)	Yes	Various masculine social norms were associated with increased odds of current suicidal thinking in univariate models (i.e., Playboy; Pursuit of Status; Self-reliance). Conformity to Risk-taking and Emotional Control norms were associated with reduced suicidal thinking. Only Self-reliance norms were significantly associated with higher ideation in multivariate models with all norms and covariates included
Quigley et al. (2017) [83]	456 11–17-year-olds from 5 Scottish high schools	Quantitative. Cross-sectional survey	Descriptive (behaviours) and injunctive social norms (permissiveness attitudes) in relation to eight reference groups (proximal-distal: from close friends to people in general) for self-harm behaviours and thoughts, and suicidal thoughts and attempts. Self-other discrepancies tested based on misperceptions of norms vs actual reported norms (SNA)	Yes	Various positive relationships between suicide/self-harm perceived norms and reported norms across outcomes (e.g., self-harm, suicide attempts, attitudes to self-harm/suicide attempts) and eight reference groups (from proximal, e.g., ‘friends’, to distal, e.g., ‘people in general’). More proximal reference groups tended to be more strongly positively associated with reported norms but this varied across norms.
Green et al. (2018) [79]	912 Students from two US universities aged 18–24 years	Quantitative. Cross-sectional survey	Conformity to masculine norms (CMNI-22)	Yes	Conformity to masculine norms (total CMNI scores) were positively associated with increased odds of chronic self-harm (6 or more lifetime instances) in multivariate models, and positively associated with a number of specific self-harm behaviours (e.g. injury via burning).
McDermott, R., et al. (2018) [93]	2504 US university students (single university)	Quantitative. Cross-sectional survey	Conformity to masculine norms (CMNI-46)	Yes	Negative associations between specific masculine norms for most CMNI subscales with intentions to seek help for suicidal thoughts from formal and informal sources. Conformity to Emotional Control and Self-reliance masculine norms had the strongest negative relationships with seeking help from informal and formal sources of support, no significant associations for help-seeking with Risk-Taking norms.
McDermott, E., et al (2018) [69]	29 (Stage 1 interviews), 789 (Stage 2 questionnaires), LGBT youth aged 13–25 years in England recruited from community youth groups, online, and via mental health services	Stage 1: Qualitative face-to-face or online semi-structured interviews. Stage 2: Quantitative online cross-sectional self-report survey.	Violating traditional gender/sexuality norms (identified in Stage 1 interviews and tested in Stage 2 quantitative study)	Partly	Qualitative: not conforming with traditional gender/sexuality norms was one of five themes associated with suicidality. Quantitative: mention of test of sexuality/gender norms but lack of detail on how these norms were measured (participants with histories of planned/attempted suicide scored higher on ’feeling more negative about gender/sexuality’)
Genuchi (2019a) [76]	94 Homeless men from one US city	Quantitative. Cross-sectional survey	Conformity to masculine norms (CMNI-46)	Yes	Self-reliance masculine norms (CMNI) were positively associated with a history of suicidal ideation versus no history. None of the CMNI norms were associated with the severity of suicidal ideation in linear regression models when including other covariates.
Genuchi (2019b) [94]	94 Homeless men in the USA	Quantitative. Cross-sectional survey	Conformity to masculine norms (CMNI-46)	Partly	Conformity to masculine norms (CMNI total scores) were not associated with suicidal ideation in multivariate analyses, but CMNI Playboy and Violence norms were positively correlated with suicidal ideation in participants reporting current ideation.
Reyes-Portillo et al. (2019) [95]	2100 US New York high school students (Grades 9–12; 13–18 years of age)	Quantitative. Cross-sectional survey (part of a larger study)	Perceived descriptive norms for suicidal ideation and suicide attempts (percentage of teenagers your age who have experienced suicidal ideation or attempted suicide in past year)	Yes	Multivariate analyses showed positive associations between personal suicidal ideation and lifetime attempts with perceived peer ideation and attempt descriptive social norms.
Chen et al. (2020) [66]	262 Veterans with depression attending a US armed forces veterans’ health facility	Quantitative. Prospective survey (12 months)	Perceived descriptive and injunctive (proximal = ’people important to you’, and distal = ’people your age’) social norms for seeking treatment for depression	No	None of the social norm measures were significantly associated with changes in suicidal ideation.
Choi et al. (2020) [96]	Filipino-American and Korean American adolescents aged 11–19 years old (Chicago, USA) Wave 1 (n = 761), 2 (n = 604), 3 (n = 641)	Quantitative. Multi-wave prospective surveys over four years	Perceptions of Asian American parents’ conformity to traditional sociocultural gender norms	No	No relationship between perceived parental norms and adolescent suicidal ideation.
Fadoir et al. (2020) [67]	185 participants admitted to USA hospitals due to recent suicidality (attempted suicide or suicidal ideation)	Quantitative. Cross-sectional survey (self-report and clinician-reported measures)	Conformity to masculine norms (CMNI) Restrictive Emotionality subscale only	Yes	Bivariate correlations: Conformity to Restrictive Emotionality norms were positively correlated with Fearlessness About Death and suicide risk for women, but not men. Multivariate models: Restrictive Emotionality was positively associated with Fearlessness About Death (accounting for various clinical covariates). Moderated mediation effect: gender moderated the mediation of Restrictive Emotionality by Fearlessness About Death on suicide risk (for women, not men).
Hill et al. (2020) [97]	3673 18–30-year-old men from 3 countries (UK = 1225, Mexico = 1120, US = 1328)	Quantitative. Cross-sectional survey (secondary analysis of existing data)	Conformity to masculine social norms (new ’Man Box Scale’ measure)	Yes	Stronger conformity to masculine norms was associated with more severe current suicidal ideation (past two weeks).
King, K., et al. (2020) [98]	26 Australian men aged 80 years and older	Qualitative (Thematic Analysis). Semi-structured focus groups (plus one 1:1 interview, six questionnaires)	Masculine social norms (specifically independence and self-reliance)	Yes	Qualitative: hegemonic masculine norms relating to independence and self-reliance discussed as part of the "reasons for suicide" theme.
King, T.L., et al. (2020) [99]	829 Australian adolescent boys and young men (aged 15–18 years at baseline; 17–20 years at follow-up)	Quantitative. Data from a larger longitudinal national multi-wave survey, two waves sampled	Conformity to masculine norms (CMNI-22)	Yes	Conformity to masculine social norms (CMNI) relating to Violence and Self-reliance were associated with higher odds of suicidal ideation in the past 12 months, whilst greater conformity to Heterosexuality Presentation norms were associated with lower odds of suicidal ideation. Other CMNI subscales were not associated with suicidal ideation.
Rezapur-Shahkolai et al. (2020) [73]	923 Iranian married women sampled based on health records (from 1 county in Iran)	Quantitative. Cross-sectional survey	Subjective norm based on the TPB (4 items with different referent groups, including the perceived importance of views about suicide for husbands, friends, religious leaders, family members)	Partly	No relationship between subjective social norms with suicidal ideation in Structural Equation Model. Bivariate analyses indicated that subjective norms were negatively correlated with suicidal ideation and intentions, and positively with suicide-related attitudes and perceived behavioural control over suicide.
Wallace et al. (2020) [100]	5131 US college students (one university in Colorado)	Quantitative (Machine Learning). Cross-sectional self-report surveys (data collected in one of four waves in 2011, 2013, 2015, 2017)	Perceived typical student (same university) descriptive norms on the use of alcohol and cannabis over the previous 30 days (Cannabis items taken in latter in 2013, 2015 and 2017)	Yes	Lower perceived peer alcohol use (descriptive norms) was associated with lifetime histories of NSSI and suicidal ideation for males (i.e. increased suicide risk). Perceived norms not a predictor of NSSI or ideation for females.
Carter et al. (2021) [82]	Adolescents and young adults aged 13–22 years from Australia/New Zealand, USA, UK, and Brazil recruited online via a global marketing company. Different samples per hypothesis (overall, n = 1624 who watched 13 Reasons Why; n = 1896 who didn’t)	Quantitative. Cross-sectional survey	Perceived same-age (adolescent) peer descriptive and injunctive norms about prevalence of anxiety, depression and suicidal ideation, and how accepting peers are perceived to be of these experiences (TNSB)	Yes	Both perceived peer descriptive and injunctive norms (prevalence and acceptability of mental health experiences) were positively associated with talking about suicide and reaching out to friends. A descriptive norm by injunctive norm interaction was found for talking about suicide and reaching out to friends (relationship between descriptive norms and outcomes was stronger as injunctive norms increases in Australia/NZ/UK/USA). Some differences between countries (e.g., negative interaction effect for reaching out to friends in Brazil)
Bock et al. (2021)* [78]	Sample A (male US undergraduate students, n = 472), Sample B (males aged 50 years and older recruited online, n = 419)	Quantitative. Cross-sectional survey.	Conformity to masculine honour norms (relating to perceived masculinity, honour, protecting family honour and reputation, and appropriateness of aggressive behaviour to protect family)	Yes	Conformity to masculine honour norms were positively associated with the capability for suicide (correlations, direct effects in mediation analysis); this effect was mediated by the experience of painful and provocative life events for both samples.
Daruwala et al. (2021)* [71]	953 Currently serving US armed forces personnel	Quantitative. Cross-sectional survey.	Conformity to masculine norms (CMNI-46 Self-reliance subscale only)	Yes	Self-reliance masculine norms were positively associated with suicide capability (fearlessness about death), thwarted belonginess and perceived burdensomeness (correlational analyses). Self-reliance only associated with thwarted belonginess and perceived burdensomeness but not suicide capability in regression analyses (adjusting for covariates, e.g., sex, age, income).
Lueck (2021)* [81]	5010 Nationally representative sample of US adults recruited online	Quantitative. Cross-sectional survey.	Descriptive and injunctive help-seeking norms for depression based on the TRA (referent group: people important to you)	Yes	Descriptive but not injunctive norms were positively associated with suicidal ideation (bivariate correlations).
Min et al., (2021)* [84]	Study 1 (657 US undergraduate students); Study 2 (Sample 1: 657 US University Students; Sample 2: 227 US adults aged 18 years and older from an online participation pool)	Quantitative. Cross-sectional survey.	Study 2 only: perceived typical student/adult descriptive (lifetime NSSI amongst typical students/adults and percentage of typical students/adults who engaged in NSSI in last month) and injunctive norms for NSSI (how acceptable and understanding typical students/adults are of NSSI)	Yes	Study 2 (only): students without histories of NSSI were more likely to believe the typical student had engaged in NSSI (descriptive norm), and perceived typical students were more understanding or neutral towards NSSI (injunctive norm), compared to students with NSSI histories. Students with NSSI had higher estimates of the percentage of female students who engaged in NSSI. No relationship between student NSSI history on perceived acceptability of NSSI (injunctive norm); both groups thought typical students would view NSSI as not being acceptable. No relationship between students’ NSSI history on overestimations of typical student NSSI (based on Study 1 norms), but evidence that students generally overestimated typical student NSSI descriptive norms compared to other studies. Study 2 (adult sample): no relationship between NSSI history with perceived descriptive or injunctive NSSI norms, but adults with NSSI thought that a greater percentage of males and females engaged in NSSI in the last month. Across student and adult samples there was a tendency to overestimate NSSI descriptive norms.
Shin et al., (2021)* [74]	984 South Korean Adults aged 19–59 years recruited online	Quantitative. Prospective (three wave) survey over 12 months.	Perceived pro-suicidal behaviour/attempts descriptive and injunctive norms (referent group: people similar/important to you)	Yes	Perceived pro-suicide injunctive and descriptive norms were positively correlated with suicide intentions (correlational analyses), but only injunctive norms were positively related to suicide intentions in the path analysis. Pro-suicide injunctive norms also mediated the relationship between communication about suicide with friends/family/co-workers and suicide intention.

Key: CMNI = Conformity to Masculine Norms Inventory; IPA = Interpretative Phenomenological Analysis; MRNI-R Male Role Norm Inventory-Revised; SNA = Social Norms Approach; TNSB = Theory of Normative Social Behaviour; TPB = Theory of Planned Behavior; Theory of Reasoned Action. References to ‘self-harm’ or NSSI reflect the authors’ wordings.

* Studies identified in the top-up searches

Seven out of the thirty-one quantitative studies explicitly tested a theoretical model of suicide in their aims and hypotheses. Six of the reviewed studies [67, 71, 75–78] tested hypotheses that were based on the Interpersonal Theory of Suicide (IPTS) [12, 27]. One study tested more general assumptions of Nock and Prinstein’s Four-Factor Model of self-harm in the context of conformity to masculine social norms [79, 80]. None of the IPTS studies fully tested the three main components of the model at the same time (i.e., thwarted belonginess, perceived burdensomeness, acquired capability); for example, Daruwala et al. (2021) measured all three constructs but only the ‘fearlessness about death’ aspect of acquired capability, not pain tolerance [71]. Four studies only measured the acquired capability for suicide in terms of the ‘fearlessness about death’ construct [67, 75, 77], with one study measuring acquired capability as a suicide-related outcome [78], and a further study measured the thwarted belongingness and perceived burdensomeness constructs but not acquired capability [76]. Ten studies tested theories featuring social norms, including: the Theory of Planned Behaviour [64, 65, 70, 73, 74]; the Theory of Reasoned Action [81]; the Theory of Normative Social Behavior [82]; the Social Norms Approach [83, 84]; with a further study testing predictions of overestimations of peer depressed mood norms in the context of Beck’s Cognitive Theory of Depression [85]. No studies tested both a theory of suicide and a theory of social norms at the same time.

### Types of social norms studied in relation to NSSI/suicidality

There was substantial variability in the types of perceived social norms investigated or explored across studies, and in their measurement. All of the quantitative studies (n = 31, plus E. McDermott et al.’s 2018 mixed methods study [69]) used some form of self-report questionnaire measure to assess perceived social norms. For the qualitative studies, perceived social norms were discussed as themes in their own right or as part of broader themes, but none of the reviewed qualitative studies included a standardised assessment of perceived norms.

Not all of the reviewed studies focused on measuring specific suicide-related norms in relation to suicide-related outcomes. Nine studies reported assessing explicit suicide-related norms, such as the perceived prevalence of suicidal behaviour or others’ attitudes towards self-harm behaviours or suicidality [64, 65, 73, 74, 82–85, 95]. Sixteen studies focused on the conformity to some form of masculine social norm (e.g., masculine gender roles, masculine sense of honour, traditional or hegemonic norms) [67, 68, 71, 75–79, 90–94, 97–99], with one study focusing on female gender roles in relation to cultural norms associated with childbirth during marriage [72], and one study focusing on the non-conformity to broader traditional gender and sexuality norms amongst LGBT youth [69]. Two studies measured norms relating to the experience of depression and anxiety-related symptoms [82, 85]. Five studies focused on help-seeking norms, including perceptions of the acceptability of seeking help for suicidality and depressed mood [66, 70, 81, 89, 91]. Two studies reported the role of perceived parental norms and beliefs on suicidality amongst adolescents, including parents’ prosocial behaviours [86] and Asian American parents’ beliefs conforming to traditional sociocultural norms about gender [96]. Finally, two studies measured the influence of substance-use related norms in relation to suicidality, including alcohol and cannabis use [88, 100]. The next section provides a summary of these studies’ findings by the type of social norms investigated, grouped by male and masculine norms, female and feminine norms, gender and sexuality norms, descriptive and injunctive norms, normative misperceptions, and other forms of perceived norms.

#### Masculinity and male norms

Most studies in the review (n = 16) focused on conformity to some form of masculine social norms. Nine quantitative studies used the 22-item [75, 79, 92, 99], 46 item [76, 93, 94], or individual subscales from the Conformity to Masculine Norms Inventory (CMNI), such as the Emotional Control [67] or Self-Reliance subscales [71]. Another four quantitative studies used other masculine norms scales, including newly developed scales [77, 78, 90, 97], whilst three qualitative studies focused on conformity to various aspects of masculine norms [68, 91, 98].

In terms of studies measuring overall conformity to masculine norms, higher total CMNI scores were associated with higher odds for a suicide attempt in the past year amongst a sample of US men who had been sexually abused as children [75], and more chronic NSSI amongst a mixed-gender sample of US college students [79]. One study reported no significant relationship between total CMNI scores and suicidal ideation amongst US homeless men in multivariate analyses, but positive correlations between conformity to Playboy and Violence CMNI subscales with ideation amongst participants reporting current suicidal ideation [94].

Several studies focused on specific CMNI norm subscales in their analyses, with Self-Reliance norms associated with current suicidal ideation amongst a large sample of Australian men when controlling for other CMNI subscales and demographic variables [92] (although other CMNI subscales were associated with ideation in univariate models). On a similar note, McDermott et al. (2018) reported negative relationships between most CMNI-46 subscales with intentions to seek formal and informal help for suicidal thoughts in a mixed gender sample of US college students, with conformity to Emotional Control and Self-Reliance norms having the strongest associations with intentions [93]. Conformity to Self-Reliance norms were significantly positively associated with current suicidal ideation in a logistic regression analysis (current vs. no current ideation), but none of the CMNI-46 subscales were significantly associated with the severity of current ideation in a sample of US homeless men [76]. Amongst a sample of young Australia men, conformity to Violence and Self-Reliance norms were associated with higher odds for suicidal ideation, but conformity to Heterosexual norms were associated with lower odds for ideation [99]. Daruwala and colleagues reported that conformity to Self-Reliance norms were associated with the Perceived Burdensomeness and Thwarted Belonginess components of the Interpersonal Theory of Suicide [12, 27] but not the Acquired Capability factor in a sample of current US military personnel [71]. Amongst recently admitted psychiatric inpatients, conformity to Restrictive Emotionality norms were positively associated with Fearlessness about Death (part of the Interpersonal Theory of Suicide’s Acquired Capability component), with a moderated mediation of Restrictive Emotionality on suicide risk through Fearlessness about Death, only for women but not men [67].

In terms of other masculine norms assessments, Hill and colleagues (2020) developed a new measure of conformity to traditional masculine norms in three international samples of young men (‘The Man Box Scale’), with higher scores on a short form of this new scale associated with higher odds for current suicidal ideation [97]. Granato et al. (2015) used the Gender-Role Conflict Scale (GCRS) [101] to predict scores on the Acquired Capability for Suicide Scale [102] amongst a mixed gender sample of US college students, with three of the four GRCS norms having significant direct effect on capability scores (Success, Power and Competition; Restrictive Emotionality; Restrictive Affectionate Behaviour between Men; but not Conflict between Work and Family Relationships), with some norms having significant indirect effects on capability via stressful life events (Success, Power and Competition; Restrictive Emotionality; Conflict between Work and Family Relationships; but not Restrictive Affectionate Behaviour between Men) [77].

Three qualitative studies explored the role of some form of masculine norm conformity in the experience of NSSI and/or suicidality. Amongst young men (aged 16–18 years) in contact with community mental health services in England, there were perceptions that men of their age should be ‘tough’ and able to cope with emotional difficulties in an independent manner [68], but changes in these traditional perceptions of masculinity were associated with increased help-seeking for self-harm and suicidality, and in turn with increased feelings of masculinity [68]. Canadian men in Oliffe et al.’s (2017) study discussed how perceptions of masculine norms of self-reliance and emotional control were implicated in their experiences of suicidality, particularly a sense that men have to be ‘stoic’ and not show evidence of emotional distress [91]. Amongst a sample of older Australian men (aged 80 years and older), masculine social norms relating to self-reliance, independence, and stoicism, were similarly discussed in relation to coping with ageing, mental health challenges, and suicidality [98]. Suicide as an act was also discussed as a means of maintaining one’s independence and retaining one’s sense of masculinity and control later in life [98].

#### Female and feminine norms

Only one of the reviewed studies solely focused on women’s and/or feminine-related social norms [72]. Jordal and colleagues’ qualitative study explored on the experiences of unmarried pregnant women and single mothers in Sri Lanka in terms of socio-cultural norms surrounding childbirth outside of marriage, with some participants becoming pregnant after rape. Mothers discussed how they were more likely to feel suicidal or attempt suicide as a result of becoming aware that they had violated local sociocultural sexual norms by becoming pregnant and having children outside of marriage, particularly through feelings of self-blame and shame [72].

#### Gender and sexuality norms

One mixed-methods study identified that a combination of gender and sexuality norms was one of several social determinants of NSSI and suicide risk amongst LGBT youth in England [69]. Specifically, perceived gender and sexuality norms which made LGBT youth feel that something was wrong with them as individuals were identified as influences on suicidality through initial qualitative interviews. A second stage quantitative questionnaire indicated that LGBT youth with histories of planned or attempted suicide were more likely to “feel negative” on these gender/sexuality norms versus those without histories of suicidal behaviours, although the specific wording of these norms assessed in the questionnaire were not explicitly stated in the reported study [69].

#### Subjective norms (Theory of planned behaviour)

Seven studies were explicitly based on the Theory of Planned Behaviour (TPB), including the related Theory of Reasoned Action and the Reasoned Action Framework [37, 103], and explicitly tested the TPB’s subjective norms concept, typically defined as the perceived behaviour or attitude of an important other or others [64, 65, 70, 73, 74, 81, 87]. In the earliest published study included in the present review, O’Connor and Armitage (2003) found that subjective norms, based on perceptions of ‘others who are important to me’ views of deliberate self-harm (measured on a single item), were not significantly associated with intentions to self-harm in a mixed sample of participants who had presented at hospital due to a recent “deliberate self-harm” episode and a hospital control sample [65]. The same study included a measure of ‘moral norms’ but these items appeared to focus more on the individual’s own personal view of “deliberate self-harm” rather than the perceived moral norms of a broader social group [65]. The same authors further tested the TPB’s revisions of the subjective norms factor, including descriptive norms (perceptions of what important others do) and injunctive norms (social approval based on perceived rewards/punishments), in a similar sample of 90 individuals who had presented at a Scottish hospital with a recent episode of “deliberate self-harm” [64]. There were no significant associations between NSSI/self-harm intentions or suicidal ideation with either norms at baseline or in prospective analyses; however, a significant descriptive norm by group identification interaction was found (shared social identity with friends/peers). For those who identified more strongly with friends/peers, intentions to engage in NSSI increased as the descriptive group norm increased [64]. O’Connor and colleagues (2009) conducted a further study using these TPB norm measures with a large sample of Scottish high school students, with perceived ‘group norms’ associated with higher odds for histories of self-harm for boys but not girls [87].

Also based on the TPB, Skogstad et al. (2006) tested the predictive role of the subjective norm factor and other constructs (e.g., perceived behavioural control), on help-seeking intentions in 527 male prisoners in New Zealand [70]. The subjective norm measure included two core items assessing general and specific others’ perceived beliefs that the participant should seek help from a psychologist if they were experiencing personal problems (with the specific item averaged across seven different referents). Both subjective norm measures (general and specific) were positively associated with help-seeking intentions for both personal problems and suicidal feelings, with the more specific subjective norm measure being more strongly associated with both intention outcomes than general referents [70]. Rezapur-Shahkolai and colleagues (2020) similarly tested the TPB and a combined subjective norm measure (perceived opinions of suicide across different referents: friends, spouse, family, and religious leaders) in a sample of 923 married women in Iran [73]. The perceived importance of others’ beliefs about suicide were negatively associated with suicide intentions and ideation in bivariate correlations, but were not significantly associated with ideation in structural equation modelling of the predictors of suicide intentions [73].

Amongst a sample of South Korean adults, perceived “pro-suicide” descriptive and injunctive norms (relating to whether “similar” others viewed suicide as a solution to difficulties and approval of suicide as a behaviour) were both positively correlated with prospective suicidal intentions [74], however only injunctive norms were associated with intentions in multivariate analyses. Perceived pro-suicide injunctive norms also mediated the relationship between communicating about suicide with family, friends and co-workers with prospective suicidal ideation six months later, suggesting that as South Korean adults discussed suicide with others this lead to stronger perceived pro-suicide injunctive norms and, in turn, higher suicide intentions if faced with a future crisis [74]. And finally, based on further refinements to the TPB, Lueck (2021) reported a weak positive correlation between past month suicidal ideation with perceived descriptive norms but not injunctive norms amongst a nationally representative sample of US adults (based on perceptions of how important individuals would think/behave) [81].

#### Descriptive and injunctive norms

Whilst descriptive and injunctive norms are a feature of the Theories of Planned Behaviour/Reasoned Action, four studies tested the role of these norms in relation to other theoretical models or without specific basis on the TPB/TRA. Based on a large scale, US state-wide, survey of high school students’ health (n = 87,349), Swahn and colleagues (2010) investigated the role of perceived adult and peer disapproval of alcohol use (i.e. an injunctive norm) on students’ histories of suicide attempts [88]. Perceptions that peers and adults disapproved of students’ alcohol use was associated with lower odds ratios for a history of suicide attempts across the sample with some sex-specific differences; perceived adult disapproval of alcohol use was associated with a reduced likelihood of attempts for boys but not girls, whilst perceived peer disapproval was associated with lower odds of attempts for girls but not boys. Perceived adult and peer disapproval of alcohol use had protective buffering roles against suicide attempts amongst students who did not personally use alcohol (i.e. reduced the strength of the alcohol use-attempts relationship); however, those students who did use alcohol and perceived that their peers disapproved of alcohol use had higher odds for suicide attempts [88].

Chen and colleagues (2020) reported no significant relationships between proximal or distal (“people important to you” and “people your age” respectively) perceived peer descriptive and injunctive norms for seeking treatment for depression with changes in suicidal ideation over one year in a sample of US military veterans [66]. Wallace et al. (2020) applied machine learning algorithms to model the factors associated university students’ lifetime NSSI behaviours and suicidal ideation based on multi-wave cross-sectional survey data [100]. Substance-use related peer norms were measured alongside a series of demographic variables, academic performance, and other health behaviours. Perceived alcohol use norms were featured in the models for male students only, with perceptions that typical peers consumed alcohol on ten or more days a month associated with lower odds of NSSI, and students who perceived that peers consumed less than 7.5 alcohol drinks when socialising/partying having higher probabilities for lifetime suicidal ideation [100].

Carter and colleagues (2021) explored the role of perceived descriptive and injunctive norms relating to prevalence of anxiety, depression and suicidal ideation, in relation to discussing suicide and reaching out to support friends amongst a large sample of adolescents and young adults from the USA, Australia and New Zealand, Brazil, and the UK, who had watched the first season of “13 Reasons Why”, a television series which featured a prominent suicide-related storyline [82]. Across all countries there were positive relationships between perceived descriptive and injunctive norms (composite scores of the percentage of other people the same age experiencing depression, anxiety, and suicide, and the perceived acceptability of these experiences) with talking about suicide with others (a composite measure of past behaviours in relation to talking to friends, parents, teachers, and a counsellor). Excluding Brazil, there were interactions between descriptive norms with age, and descriptive norms with injunctive norms, in predicting talking about suicidal ideation. Descriptive norms had a stronger relationship with talking about suicide as injunctive norms increased but with younger ages (i.e. the descriptive norms and talking about suicide relationship weakened with increased participant age) [82].

#### Normative misperceptions

Four studies investigated the role of normative misperceptions, overestimations of peer norms, or self-other discrepancies, of perceived social norms [83–85, 95] broadly in line with the Social Norms Approach [3, 48]. For example, Geisner et al. (2015) reported under-estimations of peer sadness and depression, but over-estimations for the prevalence of peer suicidality compared to actual reported norms at the same university amongst a large sample of US students (based on feelings over the previous two weeks). Across both male and female students, personally feeling suicidal was associated with higher rates of perceived suicidality in other students [85].

Quigley and colleagues (2017) investigated the role of misperceived norms (based on potential discrepancies between personal and perceived norms) for self-harm thoughts and acts, and suicidal thoughts and acts (four descriptive norms), plus attitudes or perceived permissiveness of self-harm and suicide (two injunctive norms), in a sample of adolescents from several Scottish high schools [83]. For each norm, participants were presented with a comprehensive list of different reference groups, including perceived norms for close friends, parents, extended family, same-age/sex high school students, students at the same school, high school student norms in general, people the same-age, and people in general. Significant differences between personal and perceived norms (self-other discrepancies) were reported for all descriptive and injunctive norms, but these did vary across referent groups (e.g., high school students of the same age or sex, at the same school, high school students in general, and people in general, were all perceived to be more likely to attempt suicide than personal reported norms). There was significant variation in the perceived norms associated with self-harm and suicidality outcomes, typically the perceived norms for more proximal (i.e., socially closer) referents (e.g., friends, other students) were associated with higher odds for self-harm thoughts and behaviours, and suicidal thoughts and behaviours, than socially distant referents. The role of perceived family/parent norms was less clear, although perceptions that family members were more permissive of suicide attempts were associated with a near thirty times higher risk for more positive personal attitudes to suicide attempts amongst adolescents. There was consistent evidence of discrepancies between perceived and personal norms, but variation in these discrepancies for different referent groups and differences in the predictive strength of these norms across different outcomes and referent groups [83].

Reyes-Portillo and colleagues (2019) investigated the role of perceived peer descriptive norms for suicidal ideation and attempts in personal experiences of ideation and attempts amongst a large sample of US high school students [95]. There was evidence of overestimations of the rates of peer ideation and attempt norms compared to existing data, with greater overestimations amongst girls compared to boys. Perceived descriptive norms (ideation/attempts) were associated with increases in suicidal ideation and attempts, including when sociodemographic variables and participants’ prior exposure to deaths by suicide were controlled for in the analyses [95].

Min and colleagues (2021) investigated the existence of misperceptions (overestimations) of NSSI behaviours and attitudes (perceived descriptive and injunctive norms) across two samples of US college students and adults [84]. Students with no history of NSSI had higher estimations of the rates of past year NSSI amongst typical students, and perceived typical students had more understanding attitudes to NSSI, compared to those with NSSI histories, with no difference in the perceived acceptability of NSSI between students with and without histories of NSSI. Students with histories of NSSI had higher estimations for female students’ engagement in past month NSSI than students without NSSI histories, with no group differences for perceived rates for male students. Both groups similarly overestimated rates of NSSI amongst students compared to previous studies (but no significant between-group differences). For the adult sample, there was no difference between adults with and without NSSI histories in terms of their estimates of whether typical adults had engaged in past month, year, or lifetime NSSI, but both groups similarly overestimated NSSI prevalence. Adults with histories of NSSI thought that typical male and female adults had higher rates of past month NSSI on the sex-specific items. There were no differences between adults with and without NSSI histories in terms of perceived understanding or perceived acceptability of NSSI amongst adults. Across adults and students there seemed to be perceptions amongst those with histories of NSSI that others would not approve of or understand NSSI behaviours, but mixed findings for perceived descriptive norms with students without histories of NSSI being more likely to think that typical students do engage in NSSI but no equivalent finding for the adult sample [84].

#### Other norms

Three studies assessed other forms of perceived social norms to those previously outlined, all focusing on adolescents, including perceptions of their peers’ and parents’ prosocial behaviour [86], high school students’ perceived norms about help-seeking from various referents [89], and perceptions of Asian American parents’ conformity to gendered norms and beliefs [96]. Amongst a sample of American Indian adolescents, Pettingell and colleagues (2008) reported that perceived parental prosocial behaviour norms were protective against a history of suicide attempts for boys but not girls (i.e. perceived parental prosocial behaviours were associated with lower odds of a history of suicide attempts) [86]. Pisani et al. (2012) reported positive associations between US high school students’ perceived help-seeking acceptability norms with higher odds for help-seeking (reporting ideation to an adult and seeking help), but these associations did not remain significant in multivariate models with other attitudinal and social environmental measures [89]. Pisani et al. [89] measured perceived help-seeking norms amongst students using the Help-Seeking Acceptability at School Scale [104, 105] (note that some studies excluded from this review have used this scale and combined the intentions to seek help and perceived help seeking norms subscales into one composite score). Finally, Choi and colleagues (2020) found no significant association between Asian American adolescents’ perceptions of their parents’ gendered norms beliefs with the likelihood of suicidal ideation [96].

### Summary of the relationship between social norms and NSSI/suicidality outcomes

Twenty-seven quantitative studies reported significant associations between perceived norms and self-harm/suicidality [64, 67, 70, 71, 73–79, 81–89, 92–95, 97, 99, 100]. Three studies reported significant relationships only in correlation analyses or univariate models but not in multivariate models when other predictors and clinical variables were included [73, 89, 94]. Four studies reported no significant norms and self-harm/suicidality relationship [65, 66, 90, 96].

Twenty-two quantitative reported that perceived social norms were associated with worsened suicidality and NSSI [64, 67, 71, 73–79, 81–85, 87, 88, 92–95, 97, 100]. Negative outcomes included a history of self-harm/NSSI [84], an increased risk of self-harm [79], increased suicidal ideation [76, 85], suicide intentions [74] and suicide attempts [75], and lower likelihoods of seeking support for self-harm/suicidality [93]. Specific norms implicated in suicidality and self-harm included a greater conformity to self-reliance norms associated with masculinity [71, 92, 99]. Closer, more proximal reference groups for social norms also tended to have stronger relationships with self-harm and suicidality compared to more distal groups [83]. There were some potential sex-differences amongst younger age groups, with boys appearing to be more influenced by family and parental norms than girls (e.g. [86, 88]), and girls appearing more influenced by peers and others their age [87, 88].

Eight quantitative studies reported a protective effect of conformity to perceived social norms on suicidality and self-harm outcomes, including: a lower risk of suicide [88]; reduced suicide intentions [73] (correlation analyses only); a less severe history of suicide attempts [86]; and increased intentions to seek help or support others [70, 82, 89]. Two studies reported protective effects of conformity to specific forms of masculine norms on suicidal ideation amongst male participants, including norms relating to heterosexuality [99], and risk-taking and emotional control [92].

A number of quantitative studies reported indirect relationships between social norms and self-harm/suicidality, in the form of mediated and/or moderated effects [64, 67, 74, 77, 78, 82, 88]. Although, two studies reported non-significant mediated/moderated effects involving social norms in suicidality and self-harm [73, 81]. Example indirect effects reported in the reviewed studies include Swahn and colleagues (2010) who reported a moderation of the relationship between teenagers’ personal alcohol use and history of attempted suicide by perceived peer and adult approval norms, with lower suicide risk amongst teenagers who did not use alcohol and thought family/peers disapproved of alcohol use, and higher risk amongst teenagers who used alcohol but thought friends disapproved of alcohol use [88]. Other examples of indirect effects involving social norms include perceived pro-suicide injunctive norms mediating the relationship between communicating about suicide with friends, family and co-workers with prospective suicide intentions [74], suggesting that increased exposure to discussing suicide with close social contacts leads to stronger perceptions that suicide is a socially acceptable act thereby increasing intentions. Elsewhere, the relationship between conformity to perceived masculine honour norms and increased suicide capability was mediated by the experience of painful/provocative life events [78]. Investigating indirect relationships between perceived social norms with NSSI and suicidality was not common across the quantitative studies.

In terms of the qualitative studies, a sense of violating perceived norms in terms of gender and sexuality was discussed in relation to shame and an increased risk of suicidal thoughts and behaviours amongst Sri Lankan woman who gave birth/were pregnant outside of marriage [72] and LGBT youth in England [69]. For men especially, traditional masculine norms relating to independence, self-control, and stoicism, appear to be particularly restrictive and increased suicidality [68, 91, 98]. Age also appeared to be important for how men viewed suicide as an act, with older men viewing suicide as a means of maintaining one’s independence and sense of masculinity later in life whilst also viewing suicide as a sign of weakness amongst younger men [98]. Other work suggested that challenging traditional perceptions of masculinity in younger men can lead to increased help-seeking and increased feelings of masculinity [68]. Across the qualitative studies, not conforming to perceived ideals and social norms was typically associated with suicidality through a sense of shame or failure for not meeting some perceived set standard (e.g., in terms of expectations of gender, age, and/or sexuality).

## Discussion

Perceived social norms are a key influence on various health-related behaviours. The role of perceived norms in self-harm and suicidality-related outcomes has been unclear despite NSSI and suicidality being influenced by the formation of intentions and normative pressures. The present scoping review aimed to identify the forms of perceived social norms associated with self-harm and suicidality-related outcomes and outline the role of norms in these outcomes.

Most of the reviewed studies were quantitative, using psychometric measures of perceived social norms as part of cross-sectional survey designs. There was some variability across the perceived social norms identified in the reviewed studies. Most of the studies focused on the conformity to some form of male or masculine norms (n = 16), with only one study explicitly focused on female/feminine norms [72], and a separate study investigating combined gender/sexuality norms [69]. Seven studies measured conformity to some form of subjective norm based on the Theory of Planned Behavior (including theories related to the TRB, e.g., the Theory of Reasoned Action). A further four studies measured conformity to descriptive and injunctive norms more broadly, four studies focused on overestimations or misperceptions of descriptive/injunctive norms, and the three remaining studies all studied the influence of adolescents’ perceptions of parents and other referents. It was notable that few studies measured the influence of perceived suicide or NSSI related norms (e.g. the frequency of NSSI/suicidality, or perceived attitudes or approval of NSSI/suicidality) and few studies were explicitly based on or tested an explicit theory of suicide or social norms. No studies attempted to investigate or test theories of suicide and social norms at the same time. This lack of theory-based prospective studies limits the understanding of the potential causal role of perceived norms in self-harm and suicidality, and limits theory development.

The majority of quantitative studies reported that perceived social norms were implicated in worse suicide and NSSI-related outcomes (symptoms, behaviours, and help-seeking), with eight studies reporting more protective effects of conformity to perceived norms in relation to self-harm and suicidality. The qualitative studies tended to focus on perceived social norms implicated in worse outcomes, with the shame participants associated with a violation of or non-conformity to an expected standard discussed as a contributing factor for poorer outcomes. Although, changing and reappraising perceived (masculine) social norms appeared to be associated with improvements in help-seeking for suicidality and mental health difficulties in one qualitative study [68]. Overall, there was a focus on the role of perceived social norms in exacerbating suicidality and NSSI outcomes across the qualitative and quantitative studies included in the review.

Whilst the present scoping review provides an overview of the literature on perceived social norms and their relationship with NSSI and suicidality, caution is needed in drawing definitive conclusions about the strength or direction of this relationship based on this broader scoping of the literature. The role of perceived social norms in NSSI/suicidality appears more complex than simply greater norm conformity leading to worse outcomes, with evidence that some forms of social norms may be protective against NSSI/suicidality [86] or may promote help-seeking when suicidal [70, 89]. There is also a danger in assuming that specific social norms (e.g., traditional views of masculinity or hegemonic masculinity) are always associated with negative outcomes, without considering contextual factors or the more specific facets of such norms (e.g., there is evidence that certain aspects of “traditional masculinity” can be associated with positive health outcomes [106]). In light of this, the social norms literature should consider developments from the Positive Clinical Psychology approach which argues that such psychosocial factors are not necessarily “positive” or “negative” but may have different functions and outcomes depending on the context [107]. An individual’s perception of social norms may have different effects on their NSSI and suicidality depending on the context, the individual’s personal characteristics, their affiliated social groups, and the types of norms, as well as the type of NSSI/suicidality outcome (e.g., ideation and intentions in comparison to help-seeking behaviours).

### The operationalisation of social norms

How perceived social norms are measured and defined is a challenge for the literature [108], not just in the context of suicidality but in relation to health-behaviours more broadly [3]. As highlighted in this review, there were a variety of measures and approaches to defining perceived social norms which presents a challenge for understanding the role of perceived norms in suicidality-related outcomes and in conducting meta-analyses. Many studies fail to provide adequate details of the wording of their social norms measures, such as the behaviour or attitude and/or the referent group which the norm items measure. One study in the review used a composite social norm score across different normative referent groups, which may not account for the different effects on suicidality associated with different referents (e.g. close family members versus religious leaders [73]). Other studies in the wider literature use measures which appear to combine perceived social norms with other constructs, including attitudes and intentions [104, 105].

There is still some lack of clarity about whether NSSI and suicidality is more strongly associated with more specific perceived normative behaviours or attitudes, or which reference groups are more influential (i.e., more proximal versus more distal groups). Some, but not all, of the reviewed studies reported weak-to-moderate effects of perceived social norms on suicidality and self-harm outcomes [67, 71, 78, 81, 94]. These effect sizes may reflect that perceived social norms are only a modest predictor of outcomes in themselves (although this might vary depending on the sample and norms studied), but also the complex and multifaceted causal factors implicated in suicidality and NSSI, as well as the general difficulty in predicting these outcomes [55, 109, 110].

It was notable that few studies in the review measured or discussed the role of social identification, only one study reporting a significant interaction between stronger group identification and greater social norms of self-harm in the intention to engage in deliberate self-harm amongst a clinical sample [64]. Social identification is an important moderator of the effect of perceived norms on health behaviours and may strengthen the influence of the perceived behaviours or attitudes of a social group on the individual [111, 112], particularly for behaviourally relevant social referent groups [113]. Understanding the specific social identification processes implicated in the social norms and self-harm/suicidality relationship may be important for suicide prevention efforts, particularly for informing personalised normative feedback intervention approaches featuring social norms messages (e.g., those based on the Social Norms Approach) [3].

Conformity to perceived traditional hegemonic masculinity norms in relation to suicidality was a clear focus of the reviewed studies and it was often discussed how men appear to be at a greater risk of suicide because of restrictive male gender norms. There are various issues with psychometric measures of masculine norm conformity, in particular the widely used CMNI scale [114] which has been criticised for being less of a measure of masculine norms (i.e. what others do) and more a measure of an individual’s personal behaviours, attitudes and feelings about masculinity [115]. There is also a danger of the literature assuming that gender norms have unique influences on the sexes (i.e. perceived masculine norms being only relevant to those who identify as men), in assuming the binary nature of gender (i.e. male vs female), as well as ignoring the heterogeneity of masculine identities [99]. There was some evidence in the literature reviewed here that conformity to masculine norms and the capability for suicide or intentions to seek help is not consistently moderated by biological sex, and conformity to perceived masculine social norms can influence women’s experiences of suicidality as much as men [67, 77, 93]. It might be that men are more likely to be socialised to conform to these restrictive masculine norms, but these perceived norms and standards may be influential on individuals regardless of their sex and/or gender identity [93]. There is an ongoing discussion of the complex gender politics in the suicidology field, particularly the implicit assumptions of suicide as a male behaviour and reliance on simplistic dualistic approaches to gender [116]. Greater diversity in the sampling of participants by sex and gender in studies of masculine norms and suicidality is needed, as is the move away from binary assumptions of gender in theoretical models of suicide.

### Additional research implications

Studies also varied by their use of validated psychometric assessments of ideation and behaviour, with a number of studies appearing to use single-item measures which may not be sufficiently sensitive to distinguish between different aspects of NSSI/suicidal thoughts or behaviours [66, 75, 83, 85–89, 96, 97, 99]. Other studies (e.g. [95]) included detailed assessments of passive and active suicidal ideation, thoughts about death and dying, and thoughts more specifically related to suicidal intent respectively. Whilst the present review included studies focusing on both self-harm and suicide, these two behaviours are not necessarily synonymous as many individuals may engage in self-harming behaviours without thinking about suicide. Although, the engagement in NSSI and other forms of self-injury is a potential risk factor for suicide by increasing one’s capability for suicide [117], considering evidence of heightened rates of attempted suicide amongst individuals with histories of NSSI [69, 84, 109].

There is also a need to understand the role of other social influence factors that may mediate or moderate any potential association between perceived social norms and self-harm/suicidality. As highlighted earlier, few studies reported conducting mediation or moderation analyses, and due to the variance in the types of social norms measured across studies, different forms of perceived norms appeared to have different roles in the pathways to self-harm and suicidality. More fine-tuned analyses focusing on indirect relationships would improve the understanding of the ‘how’ and ‘when’ perceived social norms are implicated in outcomes relating to self-harm and suicidality. A key factor to consider may be the exposure to self-harm or suicide in the individual’s social environment and their relationships with friends, family, and/or peers [118–120], which may influence the capability for suicide [12, 27], and inflate the perceived social norms of others’ self-harm/suicide-related behaviours and attitudes [121]. Exposure to suicide amongst social groups was not commonly measured or featured in the reviewed studies, although Reyes-Portillo and colleagues (2019) reported that perceived social norms remained a predictor of suicidal ideation whilst controlling for past exposure to suicide [95]. There may be potential interactive effects between exposure to self-harm and/or suicide in one’s social group and normative perceptions of these behaviours in relation to self-harm/suicidality outcomes. Understanding the more complex relationships between perceived social norms and self-harm/suicidality outcomes is important for developing effective intervention strategies, particularly in understanding the types of norms and mediating or moderating factors that may worsen or protect against poorer outcomes.

There is a final consideration for the broader social norms and NSSI/suicidality research literature in terms of the speculative nature of social norms’ involvement in these outcomes. As previously outlined, our literature searches identified a large body of research literature for screening, however the majority of this (then excluded) literature did not actually study ‘social norms’ as a causal factor or predictor of NSSI or suicidality. A common issue in this literature was the tendency for ‘social norms’ to offered as a general speculative causal factor for authors’ findings despite the absence of measures of social norms in the respective studies. An observation from our scoping of the existent literature is the presence of some likely common-sense assumptions of the importance and role of social norms in NSSI and suicidality, and a tendency by authors to speculate that some (unmeasured) ‘social norms’ may explain certain, potentially unexplainable, patterns of results. Greater caution is required in the literature when speculating on the role of ‘social norms’, however defined, in the experience of NSSI and suicidality.

### Implications for practice

Whilst caution should be exercised in developing interventions based on the heterogenous literature identified here, challenging perceived social norms has been a focus of a number of health interventions for other behaviours [3] and there is the potential for normative feedback to be used in suicide prevention efforts. Considering that socio-cognitive variables, such as perceived norms, may influence suicide-related outcomes over and above clinical symptoms (e.g., depression and anxiety [64]), focusing on symptom reduction alone may not be sufficient for suicide prevention. Given that perceived social norms are a key influence on intention formation, normative feedback could be an effective means of reducing intentions to engage in self-harm and suicidal behaviours and in promoting help-seeking behaviours, although how effective such feedback would be with those in acute distress is not clear.

There have been a number of suicide prevention interventions using social norms feedback. Several studies have attempted to change perceived norms, or have directly measured changes in perceived social norms, in relation to intentions to seek mental health help [122–124] or in intervening and referring others for support, including for suicidality [125–127]. There is also some evidence to suggest that individuals overestimate rates of suicidal thoughts amongst peers [124, 128], which may indicate that challenging perceived descriptive norms amongst the wider population in terms of the frequency of suicidal ideation or self-harm behaviour could be fruitful for prevention efforts. There may be opportunities to better understand the role of perceived social norms amongst those without current or past personal histories of self-harm or suicidality, given that social norms can be powerful in terms of communicating acceptable standards and expectations for individuals in social groups. This is a complex phenomenon to study as there is evidence of overestimation of self-harm and suicidality behaviours, yet still self-harm and suicidality are viewed as socially unacceptable (e.g. [84]), which may indicate complex interactions between perceived descriptive norms (behaviour frequencies) and injunctive norms (e.g. perceived acceptability). For example, there is evidence that individuals exposed to a suicide in their social networks may perceive suicide as more common, yet still somewhat incomprehensible as an act, than those not exposed [121, 129]. Such (mis)perceived social norms may increase risk amongst those exposed to self-harm and/or suicide by making suicide appear to be a more frequent behaviour than the actual social norm but lead to feelings of shame after engaging in the same behaviours which are perceived to be socially undesirable. Such complexities represent a challenge for potential interventions, especially when ensuring that social normative feedback does not have unintended negative consequences or boomerang effects, such as increasing symptoms or distress, increasing feelings of shame by violating perceived norms, or making suicidal behaviours or planning appear more frequent than the reality [130]. Although, there is evidence that boomerang effects do not occur after normative feedback for other health behaviours, such as alcohol use [131].

### Strengths and limitations

There are several strengths and limitations to consider with the present review. A systematic search of the published literature was conducted, across a range of databases and using a broad search strategy to ensure the maximum return of eligible studies. It should be noted that only published English-language studies were included in this review which may limit the findings’ generalisability. Due to the nature of the present scoping review, which aimed to map the extent of the literature, caution is needing in drawing definitive conclusions about the strength of the relationship between perceived social norms with NSSI and suicidality outcomes. In addition, considering the heterogenous nature of the reviewed studies, particularly in the operationalisation and measurement of perceived social norms, it would not appear to be appropriate to conduct a meta-analysis or qualitative meta-synthesis without a narrower, more selective, systematic review of the literature. The variety of the types of norms featured in the reviewed studies, as well as differences in the social norms measures used across studies and lack of theory-informed studies, also represents a challenge for ascertaining the role of perceived social norms in self-harm and suicidality. However, the present review has identified a number of quantitative and qualitative studies investigating the role of perceived social norms in NSSI and suicide-relevant outcomes, and has identified several key types of social norms that have been associated with these outcomes.

## Conclusions

This scoping review identified 36 studies investigating the role of some perceived social norm in relation to self-harm and/or suicidality-related outcomes. Most studies were quantitative and cross-sectional in nature and many focused on the conformity to some form of perceived masculine social norms in relation to NSSI and suicidality. Other identified norms associated with NSSI or suicidality included women’s norms in the context of childbirth, gender/sexuality norms, subjective norms, descriptive and injunctive norms, and norms relating to adolescents’ perceptions of their peers and/or parental attitudes or behaviours. Perceived social norms were generally associated with negative outcomes (i.e., increased symptoms suicidal behaviour, and reduced help-seeking), although some positive associations were reported (e.g., conformity to more positive perceived norms with increased help-seeking, reduced suicide risk). There was substantial variation across studies in the definition of social norms, and in the use of standardised measures of perceived norms and self-harm/suicidality outcomes. There was a general lack of theory-based investigations, with no studies appearing to test or explore theories of self-harm/suicidality and perceived social norms at the same time. There remains a gap in the literature for the integration of perceptions of social norms into theoretical models of self-harm and suicidality. Whilst perceived social norms may have a key role in self-harm and suicidality, the variability in how social norms are operationalised and measured across studies poses a challenge for drawing meaningful conclusions from this literature and in elucidating the role of perceived social norms in self-harm and suicidality-related outcomes. Future research needs to better define and outline how social norms are operationalised in their investigations, use more rigorous methodologies to quantitatively model the associations between norms and outcomes, and better outline when, where, and how, social norms influence the experience of self-harm and suicidality in ideation-to-enaction theoretical models.

## Supporting information

S1 File Preferred Reporting Items for Systematic reviews and Meta-Analyses extension for Scoping Reviews (PRISMA-ScR) checklist. (DOCX)

S2 File Additional details on the reviewed studies’ methodologies and social norms investigated. (DOCX)
