# Supplementary material for: The role of perceived social norms in non-suicidal self-injury and suicidality: A systematic scoping review
Source: PLoS One. 2023 Jun 23;18(6):e0286118. doi: 10.1371/journal.pone.0286118 (PMC10289472; doi:10.1371/journal.pone.0286118)
Supplement: S2 File — (DOCX) [file pone.0286118.s002.docx]

Supplementary File 2. Additional details on the reviewed studies’ methodologies and social norms investigated.

| **Authors / Year** | **Method** | | | | **Social Norms** | |
| --- | --- | --- | --- | --- | --- | --- |
|  | *Methodology* | *Primary analysis* | *Self-harm and Suicidality Measures (excluding social norms)* | *Other Measures* | *Type of norms* | *Measure used* |
| O'Connor & Armitage (2003) | Quantitative. Cross-sectional survey | Linear Regression | Main outcome: (deliberate) self-harm intentions (TPB). Other suicide-variables based on TPB (e.g., attitudes, norms, self-efficacy, personal moral norms, anticipated affect) | None | Subjective self-harm norm based on the TPB (perceived social pressure to harm oneself from perspective of 'people important to me') | Quantitative: single item measure based on standard TPB wording for norm measures |
| O'Connor et al. (2006) | Quantitative. Prospective (3 month) self-report survey | Linear Regression (including interactions) and Mediation | Time 1: frequency of past deliberate self-harm (DSH), TPB (DSH) measures (DSH intentions, affective and instrumental attitudes, self-efficacy, perceived controllability of DSH). Time 2: single items for suicidal ideation and behaviours (since T1) | Hopelessness (BHS), anxiety and depression (HADS), group/social identification | Descriptive Group Norms (perceptions of friends/peers' self-harm behaviours and others' attitudes of own self-harm) and Injunctive Social Norms (perceived important others' approval of self-harm) | Quantitative: TPB based descriptive (4 items) and injunctive (2 items) social norm self-report measures |
| Skogstad et al. (2006) | Quantitative. Cross-sectional survey | Linear Regression | Suicidal Ideation (SIQ), suicide-related TPB variables (e.g., subjective norms, attitudes, PBC, intentions), help-seeking intentions for suicidal thoughts | General and specific forms of TPB constructs, emotional distress, attitudes towards help seeking, prior help seeking with healthcare professional | Specific and general subjective norms based on the TPB (general = beliefs of people in the inmate's life about seeking help; specific = seven influential referents for help seeking) | Quantitative: novel items based on standard TPB wordings |
| Pettingell et al. (2008) | Quantitative. Cross-sectional survey | Wald Chi Square/Logistic Regression | Single categorical item for lifetime suicide attempts (yes – once; yes >2 times; no) | Various items relevant for American Indian health and wellbeing (e.g., cultural identity, relationships, decision-making), demographics. | Perceived parental and peer prosocial behaviour norms | Quantitative: novel items |
| O'Connor et al. (2009) | Quantitative. Cross-sectional survey | Logistic Regression | Single item lifetime self-harm measure | HADS, impulsivity, self-concept, trait optimism, social perfectionism, demographics. | Peer and friends 'group norms' (perceived attitudes towards self-harm) from a previous study [60] | Quantitative: group norms measures appear to be novel items. Family/friend self-harm behaviours analysed by binary outcome (yes/no) |
| Swahn et al. (2010) | Quantitative. Cross-sectional survey | Logistic Regression | Suicide attempt in past year (single item; yes/no) | Current substance use, demographics, bullying (perpetration/victim), weapon carrying, alcohol use beliefs, alcohol and drug education | Perceived peer and adult disapproval of alcohol use (injunctive norm) | Quantitative: novel items |
| Pisani et al. (2012) | Quantitative. Cross-sectional survey | Chi Square, MANCOVA/ANCOVA, Generalised Linear Mixed Models | Suicidal Ideation (Youth Risk Behavior Survey - single item, past 12 months), suicide attempts (past 12 months, single item), disclosure of suicidality, help-seeking for suicidality | Personal attitudes about help seeking for emotional distress, perceived availability of adult help for suicidality, overcoming barriers to seeking help for suicidality, depression (SMFQ), coping resources, school engagement | Perceived acceptability of seeking help for emotional distress (from perspective of friends and family) | Quantitative: Help Seeking Acceptability at School Scale |
| Easton et al. (2013) | Quantitative. Cross-sectional survey | Logistic Regression | Suicide attempt and ideation (past 12 months, yes or no) based on the General Mental Health Distress Scale | CSA (frequency, force of abuse, physical abuse), depression (Depression Symptom Scale), demographic variables (race, time since abuse, education level) | Conformity to masculine norms | Quantitative: CMNI-22 (dichotomised into high conformity of top 20% of scorers vs low conformity - others) |
| Jordal et al. (2013) | Qualitative. Cross-sectional semi-structured interviews | Qualitative content analysis | None | Demographics (e.g., age, years of schooling, occupation, planning to keep their baby or planning adoption) | Perceived cultural and societal norms of expectations of women and childbirth (i.e., giving birth to children when married) | Qualitative: norms discussed in themes relating to violating perceived sociocultural expectations of motherhood by giving birth outside of marriage |
| Geisner et al. (2015) | Quantitative. Cross-sectional survey | Correlations, Linear and Logistic Regression | Suicidal feelings (single item, past week yes/no) | BDI (depression), personal feelings of sadness/'blue' (past two weeks, novel items), perceived social norms for sadness/depression, demographics | Perceived same-campus student mood norms (percentage of students feel sad, depressed or suicidal in past two weeks) compared with actual reported norm (students then categorised into over-, under-estimating, or correct estimators) | Quantitative: Novel item |
| Granato et al. (2015) | Quantitative. Cross-sectional survey | Correlations, MANOVA, Mediation and Moderation | Acquired capability for suicide (ACSS) | Exposure to painful and provocative life events, demographics | Conformity to masculine gender role norms | Quantitative: Gender Role Conflict Scale |
| Coleman & Paggi (2017) | Quantitative. Cross-sectional survey | Factor Analysis, Correlations | Suicidal ideation and behaviours measured by the SBQ-R | Femininity ideology, depressive symptoms (Geriatric Depression Scale), alcohol abuse, demographics | Conformity to traditional masculine social norms | Quantitative: Male Role Norm Inventory-Revised (self-rated and a proxy-rated measure of masculine norm conformity) |
| Hassett & Isbister (2017) | Qualitative. Cross-sectional semi-structured interviews | Interpretative Phenomenological Analysis | None (aside from screening criteria – episode of self-harm in past 12 months) | Means of referral into health service (e.g., by general practitioner) | Conforming to and changing perceived masculine social norms | Qualitative: themes discuss how the male participants perceived masculine norms in relation to other young men (specifically conforming to muscularity/self-reliance) and how changing these norms promoted help-seeking |
| Oliffe et al. (2017) | Qualitative. Photo-elicitation interview (participants took photographs relating to their experiences of and perspectives on male suicide) | Constant comparative methods to identify themes (inductive) | Lifetime histories of suicidal thoughts, plans, and attempts | Various demographics (e.g., age, marital status, employment, mental health diagnoses) | Mixture of perceived masculine norms (control, self-reliance) and perceived general social norms about seeking help and being suicidal or depressed | Qualitative: norms discussed as part of several themes, particularly recovery from injury and having ongoing struggles |
| Pirkis et al. (2017) | Quantitative. Cross-sectional survey from first wave of a larger scale multi-wave study | Logistic Regression | Suicidal Ideation (past two weeks; single item from the PHQ-9) | Various clinical and demographic variables (e.g., age, marital status), social support, alcohol use disorders, GP use, self-reported diagnosis of depression. | Conformity to masculine norms | Quantitative: CMNI-22 |
| Quigley et al. (2017) | Quantitative. Cross-sectional survey | Friedman’s ANOVA, Logistic Regression | Lifetime personal thoughts and acts of self-harm, thoughts and acts of suicide (x4 single items), plus attitudes/acceptability of thinking about or engaging in self-harm or suicide (x4 items) | Demographics plus other health-related behaviours not reported in paper | Descriptive (behaviours) and injunctive social norms (permissiveness attitudes) in relation to eight reference groups (proximal-distal: from close friends to people in general) for self-harm behaviours and thoughts, and suicidal thoughts and attempts. Self-other discrepancies tested based on misperceptions of norms vs actual reported norms. | Quantitative: novel items based on previous SNA studies measures of self-other discrepancies/misperceptions |
| Green et al. (2018) | Quantitative. Cross-sectional survey | Logistic regression | The Deliberate Self Harm Inventory, number of people known who engage in self-harm | PANAS (mood), HANDS (depression), demographics (e.g., age, race/ethnicity, participant sex, sexuality) | Conformity to masculine norms | Quantitative: CMNI-22 |
| McDermott, R., et al. (2018) | Quantitative. Cross-sectional survey | Structural Equation Modelling | Intentions to seek help for suicidal thoughts (GHSQ) | Demographics | Conformity to masculine norms | Quantitative: CMNI-46 |
| McDermott, E., et al (2018) | Stage 1: Qualitative face-to-face or online semi-structured interviews. Stage 2: Quantitative online cross-sectional self-report survey. | Stage 1 (Constant Comparative Analysis), Stage 2 (Chi Square, Logistic Regression) | SBQ-R (suicidal ideation, plans, attempts), self-harm (yes/no), effects of abuse on suicidality | Demographics, sexuality identity, gender identity, experience of abuse related to sexual orientation/gender, stressful life events | Violating traditional gender/sexuality norms (identified in Stage 1 interviews and tested in Stage 2 quantitative study) | Qualitative: not conforming with traditional gender/sexuality norms was one of five themes associated with suicidality. Quantitative: mention of test of sexuality/gender norms but no details on how these norms were measured |
| Genuchi (2019a; J Men’s Studies) | Quantitative. Cross-sectional survey | Linear and Logistic regression | INQ (thwarted belongingness and perceived burdensomeness), suicidal ideation (BSS) | Demographics (various, including income, age, sexuality, race/ethnicity), psychiatric symptoms (PDSQ) | Conformity to masculine norms | Quantitative: CMNI-46 |
| Genuchi (2019b; Archives of Suicide Research) | Quantitative. Cross-sectional survey | Linear and logistic regression | Suicidal ideation (BSS) | Depression (Male Depression Scale, MDS; BDI), psychiatric symptoms (PDSQ), demographics (e.g., age, sexuality, race/ethnicity) | Conformity to masculine norms | Quantitative: CMNI-46 |
| Reyes-Portillo et al. (2019) | Quantitative. Cross-sectional survey (part of a larger study) | Linear and Logistic Regression | Suicidal ideation (SIQ-JR for schools, past month active and passive ideation), lifetime and recent suicide attempts (items from the Diagnostic Interview Schedule for Children), four items on exposure to suicide (family/friends/others deaths by suicide or attempts) | Demographics (age, grade, gender, race/ethnicity) | Perceived descriptive norms for suicidal ideation and suicide attempts (percentage of teenagers your age who have experienced suicidal ideation or attempted suicide in past year) | Quantitative: novel items with categorical responding based on percentages |
| Chen et al. (2020) | Quantitative. Prospective longitudinal survey (12 months) | Linear Regression | Suicidal ideation (change scores based on a single item from the PHQ-9) | Depression (PHQ-9), various social connectedness variables, number of visits to mental health and primary care services, various covariates (e.g., alcohol use disorders, post-traumatic stress, age gender, race/ethnicity) | Perceived descriptive and injunctive (proximal = 'people important to you', and distal = 'people your age') social norms for seeking treatment for depression | Quantitative: item wordings based on previous study |
| Choi et al. (2020) | Quantitative. Multi-wave prospective surveys over four years | Linear and Logistic Regression | Suicidal ideation in past 12 months (novel single item, yes/no) | Depressive symptoms (Children's Depression Inventory), various family and social functioning measures, demographics, ethnic identity and racial discrimination | Perceptions of Asian American parents' conformity to traditional sociocultural gender norms | Quantitative: 7 item scale based on previous studies |
| Fadoir et al. (2020) | Quantitative. Cross-sectional survey (self-report and clinician-reported measures) | Moderated mediation | Modified Scale for Suicide Ideation, Fearlessness About Death (ACSS-FAD) | DASS depression subscale or BDI, demographics (age, gender, ethnicity, time spent on unit, reason for admission) | Conformity to masculine norms - restrictive emotionality only | Quantitative: CMNI-Emotional Control six item subscale |
| Hill et al. (2020) | Quantitative. Cross-sectional survey (secondary analysis of existing data) | Factor Analysis and Logistic Regression | Suicidal ideation (single item from the PHQ, past two weeks) | PHQ-2 (depressive symptoms), violence-related outcomes (e.g., bullying), various demographics (e.g., age, gender, race/ethnicity) | Masculine social norms | Quantitative: development of a new measure of harmful/hegemonic masculine social norms ('Man Box Scale') |
| King, K., et al. (2020) | Qualitative. Primarily semi-structured focus groups (plus one 1:1 interview, six questionnaires) | Thematic analysis | None | Various demographics and health items (e.g., subjective health status, age, country of birth) | Masculine social norms (specifically, independence and self-reliance) | Qualitative: norms discussed in relation to 'reasons for suicide' theme |
| King, T.L., et al. (2020) | Quantitative. Data from a larger longitudinal national multi-wave survey, two waves sampled | Logistic Regression | Past 12 months suicidal ideation (single yes/no item from the Youth Risk Behavior Survey) | Various demographics and covariates (e.g., race/ethnicity, area socio-economic disadvantage) | Conformity to masculine norms | Quantitative: CMNI-22 |
| Rezapur-Shahkolai et al. (2020) | Quantitative. Cross-sectional survey | Structural Equation Modelling | Suicide ideation (BSS), TPB variables relating to suicide (intentions, subjective norms, perceived behavioural control, attitudes) | Demographics (e.g., education level, employment, history of suicide amongst friends and family) | Subjective norm based on the TPB (4 items with different referent groups, including the perceived views about suicide for husbands, friends, religious leaders, family members) | Quantitative: norms item wording based on the TPB constructs |
| Wallace et al. (2020) | Quantitative. Cross-sectional self-report surveys (data collected in one of four waves in 2011, 2013, 2015, 2017) | Machine learning based approach using Recursive Partitioning (decision tree approach) | Lifetime self-harm and lifetime suicidal ideation (analysed as binary, yes/no, outcomes) | Part of a broader survey (National College Health Assessment). Other items measured health (physical/mental), substance use, sexual behaviours, mood/affect, exercise, nutrition, demographics, identity, health behaviours | Perceived typical student (same university) descriptive norms on the use of alcohol and cannabis over the previous 30 days (Cannabis items taken in latter in 2013, 2015 and 2017) | Quantitative: norms items appear to be novel but consistent with measuring 'typical' peer behaviour |
| Carter et al. (2021) | Quantitative. Cross-sectional survey | Structural Equation Modelling | Expressing personal thoughts/feelings about suicide to others (four items: with a friend, parent, teacher, or a counsellor. Analysed as a composite score) | Demographics (e.g., gender, age, race/ethnicity), 13RW viewing, viewing of other similar TV programmes, reaching out to a friend to offer support (not specific to suicidality) | Perceived same-age (adolescent) peer descriptive and injunctive norms about prevalence of anxiety, depression and suicidality, and how accepting peers are perceived to be of these experiences | Quantitative: novel items based on the TNSB |
| Bock et al. (2021)* | Quantitative.  Cross-sectional survey. | Bivariate correlations; Mediations. | Life suicide attempts and past 12 months suicidal ideation (SBQR), suicide capability (SCS) | Firearm ownership; experience of painful and provocative life events (visual analogue scales); demographics (e.g., age, race, history of military service) | Conformity to masculine honour norms (relating to perceived masculinity, honour, protecting family honour and reputation, and appropriateness of aggressive behaviour to protect family) | Quantitative: a composite masculine honour norms conformity score combining the Honor Ideology for Manhood scale [98] and the Honor Concerns scale [99] |
| Daruwala et al. (2021)* | Quantitative.  Cross-sectional survey. | Bivariate correlations; Linear regressions. | Fearlessness About Death (ACSS-FAD), Thwarted Belonginess and Perceived Burdensomeness (INQ) | Demographics (e.g., age, sex, race/ethnicity), impulsivity, stoicism, verbal/physical aggressiveness | Conformity to masculine norms (self-reliance) | Quantitative: Self-reliance subscale from the CMNI-46 |
| Lueck (2021)* | Quantitative.  Cross-sectional survey. | Bivariate Correlations, ANCOVA, Linear Regression | Suicidal ideation (ASIQ 25 item scale) | Depression (PHQ-9), attitudes towards and intentions to seek help, perceived behavioural control and perceived capacity based on the TRA; self-stigma; current general help-seeking; job loss or income reduction during the COVID pandemic | Descriptive and injunctive help-seeking norms for depression based on the TRA (referent group: people important to you) | Quantitative: novel items consistent with TRA wording for injunctive and descriptive norms (1 item per norm) |
| Min et al., (2021)* | Quantitative.  Cross-sectional survey. | Chi Square Tests, t-tests. | Study 1: Single item measuring frequency of NSSI (past month, past year, lifetime). Study 2: types and frequency of self-injury (Inventory of Statements about Self-Injury) | Demographics (e.g., age, gender, race/ethnicity, year of study) | Study 2 only: perceived typical student/adult descriptive (lifetime NSSI amongst typical students/adults and percentage of typical students/adults who engaged in NSSI in last month) and injunctive norms for NSSI (how acceptable and understanding typical students/adults are of NSSI) | Quantitative: descriptive norm items based on the Drinking Norms Rating Form wording [100], novel items for injunctive norms |
| Shin et al., (2021)* | Quantitative. Prospective (three wave) survey over 12 months. | Path Analysis (including moderated effects) | Communication about suicide (two items measuring communication with family/friends and healthcare staff about suicide), personal pro-suicide attitudes, suicide self-efficacy; suicide intention (all novel items) | Perceived social support; demographic variables (e.g., age, gender, household income) | Perceived pro-suicide descriptive and injunctive norms (referent group: people similar/important to you) | Quantitative: novel items |

Key: 13RW: 13 Reasons Why; ACSS = Acquired Capability for Suicide; ACSS-FAD = Acquired Capability for Suicide Scale-Fearlessness About Death; ASIQ = Adult Suicidal Ideation Questionnaire; BDI = Beck Depression Inventory; BHS = Beck Hopelessness Scale; BSS = Beck Scale for Suicidal Ideation; CMNI = Conformity to Masculine Social Norms Inventory; CSA = Childhood Sexual Abuse; DASS = Depression, Anxiety, Stress Scale; DSH = Deliberate Self-Harm; GHSQ = General Help-Seeking Questionnaire; HADS = Hospital Anxiety and Depression Scale; HANDS = Harvard Department of Psychiatry/National Depression Screening Day Scale; INQ = Interpersonal Needs Questionnaire; MDS = Masculine Depression Scale; NSSI = Non-suicidal Self-Injury; PANAS = Positive and Negative Affect Schedule; PBC = Perceived Behavioral Control; PDSQ: the Psychiatric Diagnostic Screening Questionnaire; PHQ = Patient Health Questionnaire; SBQ-R = Suicide Behaviors Questionnaire-Revised; SCS = Suicide Capability Scale; SIQ = Suicidal Ideation Questionnaire (JR = junior version); SMFQ = Short Mood and Feelings Questionnaire; SNA = Social Norms Approach; TNSB = Theory of Normative Social Behavior; TPB = Theory of Planned Behavior; TRA = Theory of Reasoned Action.

*Study identified from the top-up searches.

Note that studies varied in their reference to NSSI or DSH, we have reported the authors’ used terminology in this table.
